# Assessment of community‐managed blood glucose control in patients with diabetes mellitus in Shenzhen, China

**DOI:** 10.1111/1753-0407.13573

**Published:** 2024-06-26

**Authors:** Lihua Fang, Qingxian Li, Jie Ning

**Affiliations:** ^1^ Department of Endocrinology Shenzhen Longhua Central Hospital Shenzhen Guangdong Province China

Diabetes mellitus, predominantly type 2, is a growing epidemic with nearly 90% of the cases reported worldwide.^1^ In China, community health management is pivotal for the vast number of chronic diabetes patients, directly influencing their health outcomes. Despite the importance of blood glucose control in preventing diabetes complications, many community health service centers have yet to implement effective management programs.

This study conducted a follow‐up investigation on 359 patients with type 2 diabetes managed by the Songyuan Community Health Center in Shenzhen. Data on medication, blood glucose monitoring, glycated hemoglobin, body mass index, diet, and exercise habits were collected over 6 months. Standardized management was defined by the establishment of health records and quarterly interviews, with follow‐up visits and an annual physical examination.

The study found that 62.70% of patients met the standard for blood glucose control. Univariate Chi‐square analysis identified standardized management, disease duration, and record‐keeping duration as significantly associated with blood glucose compliance rates. Multivariate logistic regression analysis, after adjusting for disease duration, record‐keeping duration, sulfonylurea therapy, and moderate exercise, revealed standardized management, medication selection, dietary patterns, and metformin dosage as significantly correlated with blood glucose control as shown in Figure 1.

**FIGURE 1:**
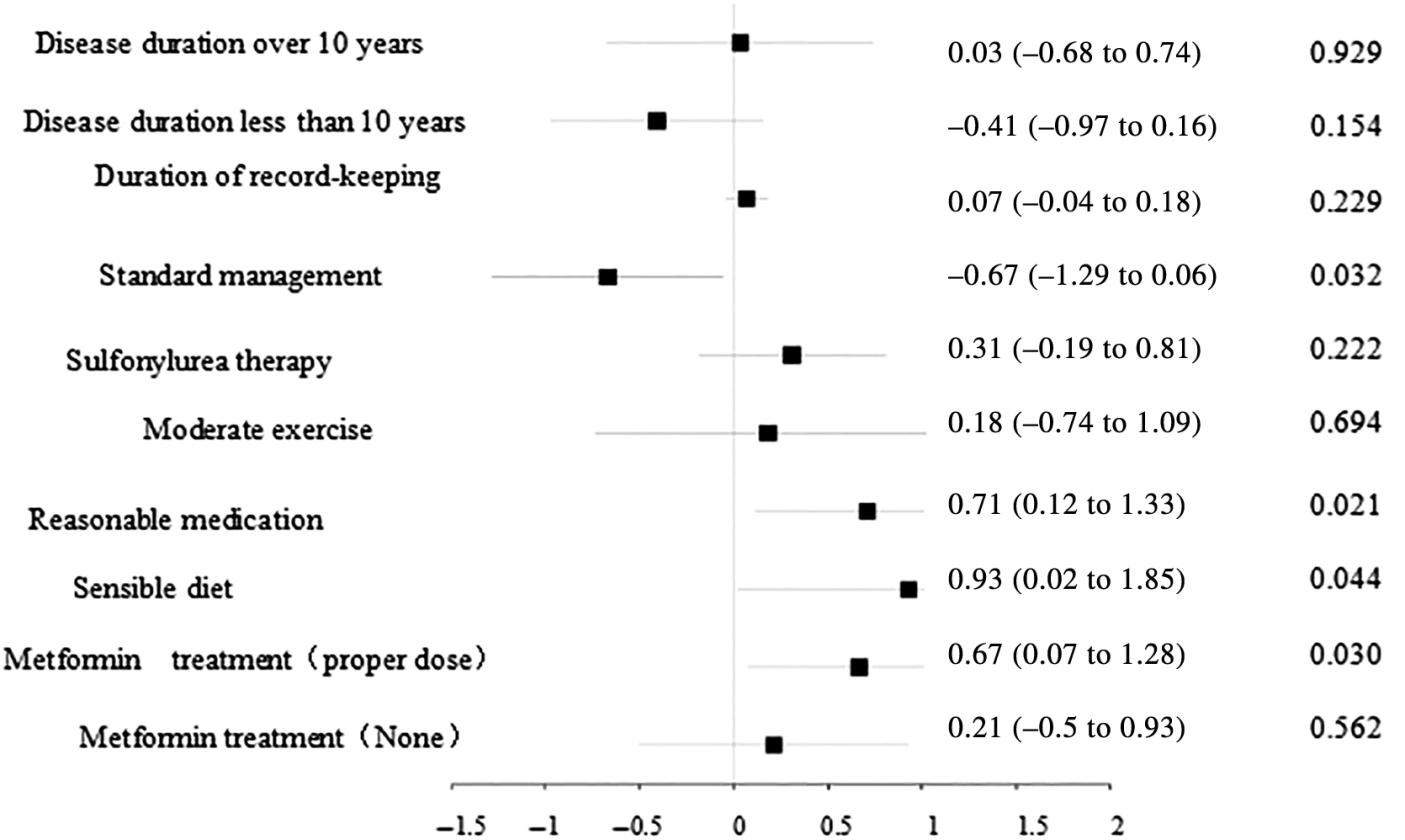
A forest diagram illustrating the results of a logistic regression analysis concerning the factors influencing blood glucose control. These factors encompass the duration of the disease, the establishment of a disease management plan, adherence to normative care practices, the use of sulfonylureas, challenges related to physical activity, choices in medication, dietary issues, and the implementation of metformin therapy. The analysis indicates that a disease course of less than 10 years and adherence to standard management practices are the most significant predictors of improved blood glucose control, overshadowing the impact of the other variables presented in the diagram.

The findings emphasize the importance of standardized management, sensible diet, and reasonable medication in improving blood glucose control among community diabetic patients. The study also highlights the need for individualized treatment strategies, particularly with metformin dosage, to achieve better glycemic management. Community health education and personalized strategies can effectively improve blood glucose control among diabetic patients. The role of standardized file management in patient management is introduced, emphasizing the need for community management systems to improve patients' blood glucose compliance rates.

This study presents novel insights into the low blood glucose compliance rate among community diabetic patients in Shenzhen, identifying standardized management, sensible diet, and reasonable medication as the primary factors influencing blood glucose control. It underscores the critical role of community health management centers in chronic disease management and the need for interventions targeting the improvement of diabetes management practices.

## CONFLICTS OF INTEREST STATEMENT

The authors have no financial or proprietary interests in any material discussed in this article.

## References

[1] Ahmad E , Lim S , Lamptey R , Webb DR , Davies MJ . Type 2 diabetes. Lancet. 2022;400(10365):1803‐1820. 36332637 10.1016/S0140-6736(22)01655-5

